# Demand Analysis of a Psychiatric Emergency Room and an Adolescent Acute Inpatient Unit in the Context of the COVID-19 Pandemic in Madrid, Spain

**DOI:** 10.3389/fpsyt.2020.557508

**Published:** 2021-01-27

**Authors:** Mónica Díaz de Neira, Hilario Blasco-Fontecilla, Lourdes García Murillo, Ana Pérez-Balaguer, Leticia Mallol, Azul Forti, Pablo Del Sol, Inmaculada Palanca

**Affiliations:** ^1^Hospital Universitario Puerta de Hierro Majadahonda, Madrid, Spain; ^2^Fundación para la Investigación Biomedica, Hospital Universitario Puerta de Hierro Majadahonda, Majadahonda, Spain

**Keywords:** COVID-19, adolescent, mental health, emergency room, acute inpatient unit

## Abstract

**Introduction:** COVID-19 represents a serious threat to mental health worldwide. The aim of this study is to identify changes in adolescent psychiatry treatment demand in a tertiary hospital in Madrid during the first month (March 11 to April 11) after the pandemic declaration by the World Health Organization (WHO). We hypothesized that fear of contagion within COVID-19 may deter people from asking for psychiatric care.

**Method:** The current study is retrospective, observational, and transversal. We reviewed the clinical records of 89 adolescents who went to the Emergency Room (ER) or were hospitalized at the Acute Inpatient Unit (AIU) at the Puerta de Hierro University Hospital-Majadahonda (PHUH-M) between March 11 and April 11. Socio-demographic, clinical, and demand variables were included in the study. Chi-square or Fisher exact tests were performed to compare categorical variables. We used the U Mann-Whitney test to compare quantitative variables. The level of statistical significance was set at p< 0.05. Analyses were conducted using SPSS v11.0.

**Results:** The number of adolescents demanding psychiatric care at the ER dropped from 64 adolescents in 2019 to 25 in 2020. Similarly, psychiatric demand collapsed from 31 to 18 patients when comparing 2019 and 2020. Furthermore, the average hospital stay in 2020 trended toward a decrease when compared to 2019 (8.94 ± 4.87 vs. 14.32 ±10.23, *p* = 0.08). Self-injurious thoughts and behaviors were the most predominant reasons for consultation at both ER and AIU.

**Conclusion:** The demand for adolescent psychiatric care decreased in the first month after the declaration of the pandemic. Our findings may be explained by (1) the fear of contagion, (2) the strict confinement measures, and (3) the initial shock as an adaptive reaction described in other disasters. Further studies are needed.

## Introduction

COVID-19 represents a serious threat to health worldwide, potentially impacting global mental health (1). Since the first COVID-19 cases confirmed in Madrid (Spain) on February 24, 2020, Madrid has become the most affected region in Spain. On the same day as the declaration of the COVID-19 pandemic by the World Health Organization (WHO) (March 11), the Autonomous Government of Madrid declared school closures. Furthermore, the Spanish government declared a national state of emergency on March 14 (Royal Ordinance 463/2020, https://www.boe.es/eli/es/rd/2020/03/14/463) including strict social distancing policies for more than 47 million Spaniards: home confinement, school closure, workplace closure, and travel restrictions, among others.

For a myriad of reasons after the outbreak, the health care system was urged to make decisions to avoid the collapse of health resources. Spain has a public health care system that covers all population health needs. In the initial stage after the outbreak, hospitals reorganized their spaces, wards, and emergency rooms (ERs) to attend mostly COVID-19 patients. In Madrid, the number of beds in adolescent acute psychiatric inpatient units (AIUs) was reduced from 73 to 41 and located at two hospitals. Furthermore, most face-to-face interventions (mental health visits, group therapy, day hospital, etc.) were canceled and immediately converted into either online or telephone call interventions.

Immediate psychological impact on mental health among the general population in China was reported during the initial stage of the COVID-19 pandemic (2, 3). In addition, behavioral and emotional disorders have been described in children and adolescents affected by the pandemic (4, 5). In light of previous health disasters, mental health consequences are presumed to be significant and long-lasting (6). Some groups may be more vulnerable than others, in particular, the pediatric population with preexisting psychiatric disorders (3, 7, 8). Despite the potential major impact on adolescent mental health (9), there is comparatively less literature about the impact of COVID-19 on both the mental health and the demand for psychiatry services in adolescents. Furthermore, the scarce literature available has been devoted to means of preventive measures to protect children, particularly those more vulnerable, against the impact of pandemics (10–12). Thus, although there is an increasing number of studies regarding several adult psychiatric aspects about the COVID-19 pandemic (3, 13–17), there is virtually no literature addressing the impact of COVID-19 on mental health services demand and the delivery of mental health care in adolescents.

The aim of this study is to analyze the impact of the COVID-19 pandemic on mental health demand from patients aged 17 or less at the ER and AIU of a tertiary hospital in Madrid (Spain) during the first month (March 11 to April 11) after the pandemic declaration by the WHO. We hypothesize that the demand for psychiatric care at both ER and AIU will decrease due to fear of contagion.

## Materials and Methods

### Sample and Procedure

The current study is a retrospective, observational, and transversal study. Eighty-nine children and adolescents aged 17 or less who went to the ER and/or the AIU at the Puerta de Hierro University Hospital (PHUH-M) between March 11 and April 11 were included. The PHUH-M is a tertiary, general hospital with 613 beds for hospitalization; it provides free, universal medical coverage to a catchment area of nearly 236,847 in the pediatric population (0–17 years old), of which 83,433 are adolescents (12–17 years old). The adolescent AIU has 10 beds for adolescents requiring psychiatric hospitalization.

ER records and psychiatric admissions in the AIU's daily record were reviewed to identify all patients. In order to ensure the objectivity of the data collected, clinical records were reviewed by two independent researchers, also by the clinicians working at the AIU during the time the data were reviewed. Whenever the information retrieved did not match, a consensus was reached with a third investigator.

The study was approved by the PHUH-M ethics committee.

### Measures

The following sociodemographic variables were included in the study: age, sex, and mental health hospitalization referral area. At the ER, we also considered the following clinical variables: reason for consultation, the International Classification of Mental and Behavioral Disorders, version 10 (ICD-10) Axis I diagnosis, antecedents of previous mental health contact, whether the patient went to the ER voluntarily or not, and attitude to discharge. The following clinical variables were also recorded at the AIU: reason for consultation, main ICD-10 Axis I diagnosis, antecedents of previous mental health contact, previous psychopharmacological treatment, previous psychiatric hospitalization at the AIU (re-admission), and days of hospitalization. We also recorded the number of all psychiatric ER patients evaluated and the occupancy rate at the AIU. Furthermore, in 2020 we also recorded data regarding the COVID-19 situation: reason for demand at the ER, and COVID-19 testing at the AIU, if available. Fear of contagion and “initial shock” were identified within clinical records as reported by patients during anamnesis at ER and admission at AIU.

### Statistical Analysis

We performed descriptive analyses using the relative and absolute frequencies for the categorical variables and the mean (± standard deviation, SD) or the median (25th and 75th percentiles), and the minimum and maximum values of the numerical variables. Chi-square or Fisher exact tests were performed to compare categorical variables. We used the U Mann-Whitney test to compare quantitative variables. The level of statistical significance was set at *p* < 0.05. Analyses were conducted using SPSS v11.0.

## Results

**Figure 1:**
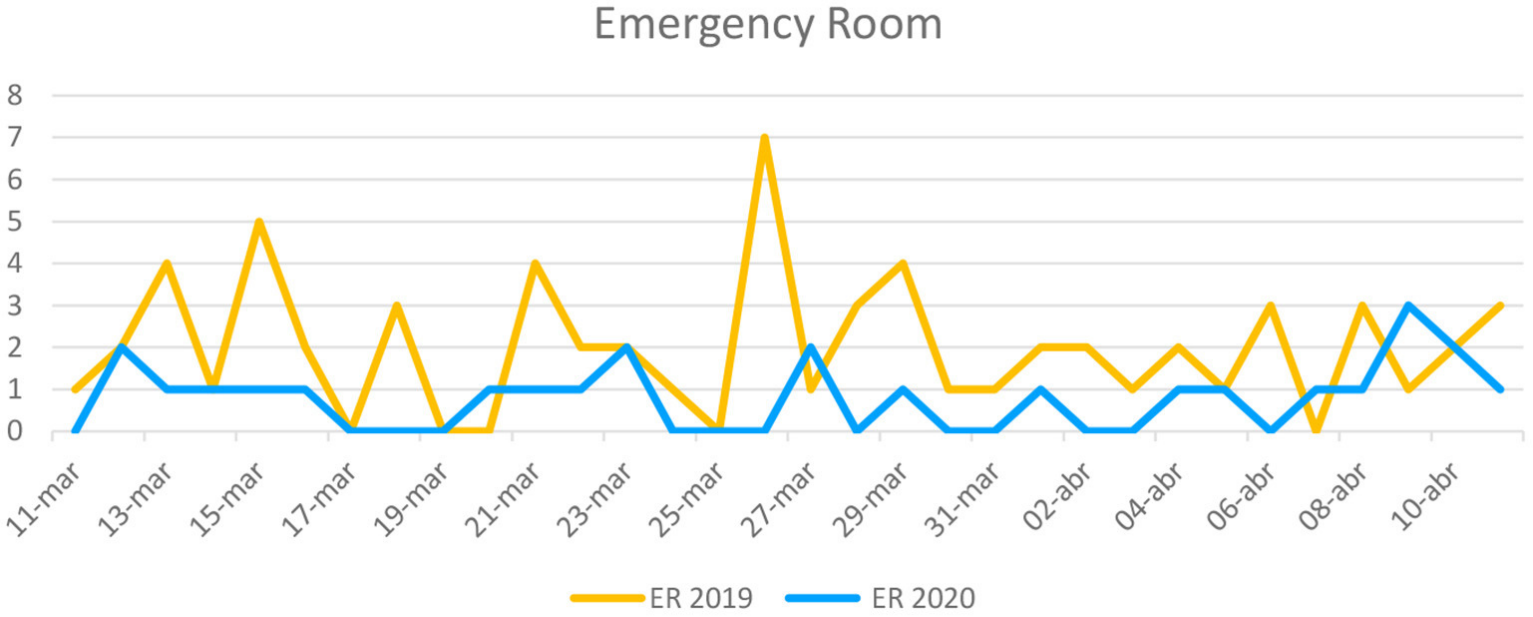
Demand for psychiatric care at the ER March 11–April 11.

**Figure 2:**
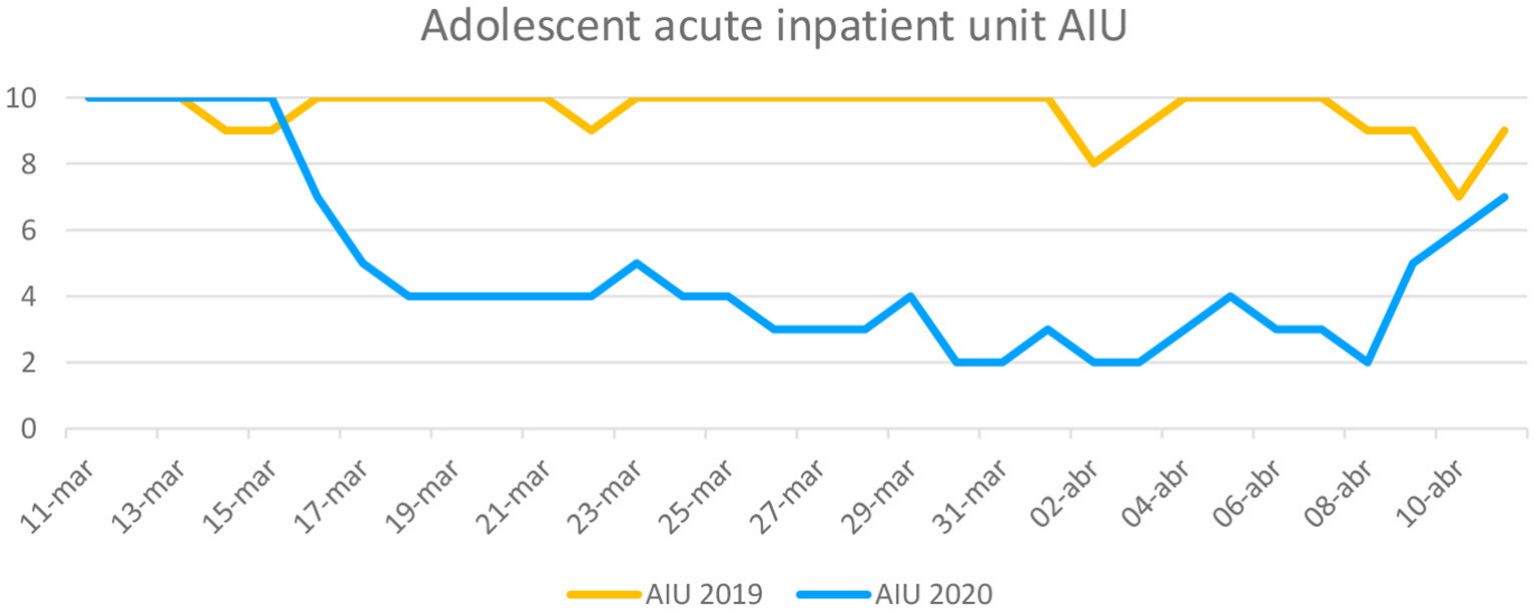
Number of adolescents hospitalized at the AIU.

Figures 1, 2 display the demand for psychiatric ER care, and the AIU's bed occupancy rate, respectively, in 2019 and 2020. Sixty-four and 25 children or adolescents were evaluated at the psychiatric ER in 2019 and 2020, respectively.

Table 1 shows the characteristics of the children or adolescents psychiatrically evaluated at the ER in 2019 and 2020. Six patients (24%) of those who went to the ER referred to the COVID-19 pandemic as the reason for their consultation.

**Table 1:** Characteristics of the children or adolescents psychiatrically evaluated at the ER.

	**2019 *n* = 64**	**2020 *n* = 25**	**Significance**
Female gender	62.5%	72%	ns
Age	14.2 (±2.3)	15.36 (±1.8)	0.09^*^
Area of referral	78.1%	64%	ns
Antecedents of mental health follow-up	90.6%	88%	ns
Voluntarily attending	78.1%	72%	ns
Hospitalization at the AIU as a result of ER consultation	28.1%	60%	**0.007**

Bold values represent significant p-values. Bold with asterisks

(*) *represent Marginally non-significant*.

Regarding hospitalization, 31 and 18 adolescents were hospitalized at the AIU in 2019 and 2020, respectively. Among the 18 patients hospitalized at the AIU in 2020, we could screen for the COVID-19 virus in nine patients; one tested positive, developing pneumonia 3 days after admission that was treated in our ward (see Table 2). The patient with the COVID-19 diagnosis was admitted in week four in an isolated room. Prior admitted AIU patients and ER patients didn't know this fact.

**Table 2:** Characteristics of adolescents hospitalized at the AIU.

	**2019** ***n* = 31**	**2020** ***n* = 18**	**Significance**
Female gender	64.5%	72.2%	ns
Age	15.55 (±1.23)	15.17 (±1.54)	ns
Hospitalization days at the AIU	14.32, 10.23	8.94, 4.87	0.082^*^
Antecedents of mental health follow-up	87.1%	100%	ns
Previous psychopharmacological treatment	67%	94.4%	**0.038**
Previous AIU hospitalization	25.8%	61.1%	**0.032**
Area of referral	41.9%	83.3%	**0.007**

Bold values represent significant p-values. Bold with asterisks

(*) *represent Marginally non-significant*.

Table 3 displays the reason for referral to the ER and hospitalization at the AIU in 2019 and 2020. Table 4 shows Axis I ICD-10 diagnoses at the ER and AIU in 2019 and 2020.

**Table 3:** Reason for referral at the ER and hospitalization at the AIU in 2019 and 2020.

	**ER 2019** ***n* = 64**	**ER 2020** ***n* = 25**	**AIU 2019** ***n* = 31**	**AIU 2020** ***n* = 18**
Anxiety	12.5%	12%	3.2%	0
Non-aggressive conduct disorder	9.4%	14%	0	0
Heteroaggression	26.6%	20%	16.1%	22.2%
Suicide attempt	12.5%	24%	22.6%	22.2%
Non-suicidal self-injury (NSSI)	12.5%	12%	0	0
Suicidal ideation	10.9%	24%	29%	44.4%
Depressive symptoms	3.1%	0	6.5%	5.6%
Psychotic symptoms	3.1%	0	9.7%	5.6%
Somatic symptoms	4.7%	0	0	0
Eating disorder	3.1%	0	12.9%	0
Other	1.6%	4%	0	0

**Table 4:** ICD-10 diagnoses at the ER and the AIU in 2019 and 2020.

	**ER 2019** ***n* = 64**	**ER 2020** ***n* = 25**	**AIU 2019** ***n* = 31**	**AIU 2020** ***n* = 18**
F10–F19Mental and behavioral disorders due to psychoactive substance use	1.6%	14%	3.2%	0
F20–F29Schizophrenia, schizotypal and delusional disorders	4.7%	0	6.5%	0
F31Bipolar affective disorder	3.1%	0	3.2%	0
F32Depressive episode	12.5%	16%	6.5%	27.8%
F41Other anxiety disorders	6.3%	0	3.2%	0
F43.1Post-traumatic stress disorder	4.7%	4%	3.2%	16.7%
F43.2.Adjustment disorders	14.1%	0	9.7%	0
F40–F48Neurotic, stress-related and somatoform disorders (F41, F43.1, and F43.2 excluded)	9.4%	0	0	0
F50Eating disorders	4.7%	4%	12.9%	0
F84.0Childhood autism	6.3%	0	16.1%	5.6%
F90Hyperkinetic disorders	4.7%	4%	0	5.6%
F91Conduct disorders	4.7%	4%	0	5.6%
F92Mixed disorders of conduct and emotions	10.9%	24%	0	22.2%
F93Emotional disorders with onset specific to childhood	0	0	29%	5.6%
F98Other behavioral and emotional disorders	6.3%	28%	0	1.2%
F94.1 and F94.2Attachment disorder	9.4%	12%	6.4%	0

## Discussion

The COVID-19 outbreak represents a serious health issue with a potentially huge deleterious impact on adolescent mental health. Some have suggested that we will see a secondary mental health pandemic due to either the direct COVID-19 impact or to the strict quarantine polices deployed by governments. In this context, we detected a significant decrease in the demand for adolescent psychiatric care both at the ER and the AIU during the first month in the immediate aftermath of the COVID-19 outbreak, as compared with the same period in 2019. The number of patients who demanded psychiatric care at the ER dropped from 64 (62% women) in 2019 to 25 (72% women) in 2020. At the AIU, the psychiatric demand collapsed from 31 (64.5% women) patients to 18 patients (72.2% women) when comparing 2019 and 2020. This sharp decrease coincides with the maximum increase in general hospital COVID-19 admissions in the Madrid community. Furthermore, the average hospitalization stay at the AIU decreased in 2020 when compared to 2019. Self-injurious thoughts and behaviors were the most predominant reasons for consultation at both ER and AIU during the COVID-19 outbreak.

Large-scale human disasters such as war conflicts, terrorism, natural disasters, or global pandemic diseases can cause a wide range of mental disorders (18). Accordingly, one might expect an increase in demand for psychiatric care in the immediate aftermath of these so-called catastrophes. For instance, Madrid's March 11, 2004, terrorist attacks produced a significant increase in demand for outpatient psychiatric care during the month after (19). However, in keeping with our hypothesis that fear of contagion to COVID-19 may deter people from asking for psychiatric care, we found that adolescent psychiatric demand collapsed in the first 3 weeks after the WHO COVID-19 Pandemic Declaration. A decrease in adult psychiatric admission rates during the lockdown period in Lombardy was also reported by Clerici et al. (15).

In the aftermath of a catastrophe, disbelief, fear, and shock are the initial responses of people (20). But compared to other human disasters, global pandemics are characterized by fear of contagion. Fear of contagion may modulate psychiatric demand as both patients and their relatives perceive hospitals as risky places for contagion. Indeed, COVID-19 fears probably kept people away from hospitals. Moreover, in this context, caregivers can be emotionally shocked and less sensitive to children and adolescent emotional needs, not taking their adolescents to the mental health services that they would use in normal circumstances (21). A second explanation for our finding of decreased adolescent psychiatric demand is that greater difficulty in accessing mental health resources (appointment cancellations, indirect telephone visiting with parents, etc.) and social services may have prevented adolescents suffering risky situations at home from asking for help (3, 22). A third explanation is that spending more time with caregivers may have had positive self-regulatory properties at home, resulting in a short-term decrease in psychopathology. Finally, school closures may have been a relief for the most vulnerable adolescents experiencing bullying, peer conflicts, or any other school-related anxiety situation, decreasing their needs for mental health care. In addition, new technologies promoting social connectedness may have also played a protective role (23).

After the initial phase, if prolonged, the quarantine may progressively increase family conflict, emotional discomfort, isolation from peers, and pessimistic thoughts, thus being a risk factor for psychiatric decompensation. Indeed, we observed a sharp increase in psychiatric demand both at the ER and AIU in the fourth week of the period studied. After the initial shock and fear due to the outbreak, quarantine and strict home confinement may have negatively impacted both adolescents and their relatives, and may have worsened pre-existing mental health disorders in children and adolescents (11, 24). Psychological impact on parents and youth associated with health emergencies has been reported (25), and parenting is challenging under these conditions. Indeed, violence and vulnerability rise during school closure periods (26). Furthermore, school closure represents a major source of stress given the drastic change in regular daily routines and the abrupt disruption of psychological support for children with special education needs usually delivered at school. For them, quarantine is particularly more stressful (10). Alongside with home confinement, children and adolescents may face their own illness or the illness or even loss of loved ones, and the economic impact of the crisis in their families (27).

Another finding is that the rate of adolescent re-admission was significantly higher during the COVID-19 crisis than in 2019. One possible explanation is that previous knowledge of the AIU together with a conflictive family dynamic at home during confinement may have facilitated asking for help at the hospital. Furthermore, it may also suggest that the most severely ill patients are those who demanded psychiatric care under these circumstances. Indeed, a significantly higher proportion of patients were hospitalized after ER consultation in 2020 when compared to 2019.

We also found that the average length of stay at the AIU decreased in 2020 when compared to 2019. This is in consonance with other reports that identified early discharge from adult psychiatry inpatient units during the pandemic (3) but in contrast with data reported by Clerici et al. (15), who reported a longer length of stay in Lombardy. This decreased AIU stay may be explained by the more restrictive conditions that governed AIU functioning following a strict COVID-19 preventive protocol, including (1) an isolation period of 48 h soon after hospitalization; (2) cancellation of family visiting and family-accompanied therapeutic outings, suspension of usual group activities (group psychotherapy, occupational therapy, community activities); and (3) the mandatory use of masks. These COVID-19 prevention measures undertaken in our unit were similar to those reported in adult inpatient settings by other authors (28). At discharge, we kept the usual protocol of community mental health care, despite existing difficulties in the context of the COVID-19 pandemic, also reported by other authors (3, 29).

Finally, self-injurious thoughts and behaviors were the most predominant reasons for consultation at both the ER and AIU. The first suicides in the COVID-19 era have already been reported in the adult population (30, 31). Indeed, some authors fear a suicide pandemic (32), particularly in the United States, given their firearm accessibility (33). Social connectedness and other preventive measures have been suggested to prevent suicides (23, 34, 35). We also found that major depression and mixed disorders of conduct and emotions were more frequently diagnosed both at the ER and AIU in 2020 than in 2019. This finding is in keeping with findings reported in China (36). These authors reported an elevated rate of affective disorders in children and adolescents during the COVID-19 outbreak.

The major strength of the current study is that our study is a “real world” observational study. Plus, the PHUH-M AIU is the only AIU in Madrid having data that allows us to review comparable situations in 2019 and 2020. To our knowledge, this is the first report of a demand analysis of an adolescent ER and AIU during the COVID-19 pandemic. However, our study has several limitations. The major limitation is the small sample size as we have only analyzed data from the first month since the COVID-19 pandemic was declared. This limitation prevented us from testing statistical differences in the prevalence of the different mental disorders between 2019 and 2020. In any case, further data will be recorded in the following months so we will be able to measure the pandemic's impact on demand once the outbreak is controlled and de-escalation of confinement is ongoing. The subjective striking observation by our team of a dramatic drop of adolescent demand for both ER and AIU services led us to study this short-term and acute phase of the pandemic in Madrid. Furthermore, given that our study is limited to the population at one geographical areas of the community of Madrid, our results may not be generalized to either other communities in Spain or other countries.

In view of the severity and length of the COVID-19 pandemic situation, future studies should confirm our initial results on the analysis of the demand of adolescent mental health services (16). We agree with the suggestion concerning the necessity of adapting the conditions of psychiatric hospitalizations to the brand new COVID-19 pandemic ecosystem (28). The incorporation of technology to improve access and quality of care of patients with mental disorders represents an opportunity for digital psychiatry and may contribute to these aims (18, 37).

## Conclusion

1. Adolescent mental health care demand decreased in the first month after the COVID-19 outbreak in the community of Madrid (Spain). Fear of contagion may partially explain this decreasing demand. Also, lockdown and social distancing measures taken may have had a protective effect in some adolescent disorders.
2. Average inpatient length of stay shortened during the pandemic. The COVID-19-preventive measures adopted during hospitalization could explain this finding.
3. Adolescents with pre-existing mental disorders are a population vulnerable to the worsening of their mental disorders as a consequence of the pandemic. Follow-up of their mental well-being must be done.

## Data Availability Statement

The raw data supporting the conclusions of this article will be made available by the authors, without undue reservation.

## Ethics Statement

The study was approved by the PHUH-M ethics committee.

## Author Contributions

IP: leadership, writing introduction, and discussion. MD: design and results analysis. HB-F: review and contribution to discussion. LG: review and contribution to results analysis. AP-B and PD: recollected data. LM: contributed discussion. AF: analysis results. All authors contributed to the article and approved the submitted version.

## Conflict of Interest

In the last 2 years, HB-F has received lecture fees from Takeda. He is the recipient of a MINECO grant (2019–2021). In 2019, he was the recipient of a PHUH intensification grant. He has also received funding from a clinical trial sponsored by Janssen (ESKETINSUI2002). IP has received lecture fees for Takeda and Rubio. She has received funding from two clinical trials sponsored by Janssen and Servier. The remaining authors declare that the research was conducted in the absence of any commercial or financial relationships that could be construed as a potential conflict of interest.
